# Dural Sealants in Spine Surgery: A Scientometric Analysis

**DOI:** 10.7759/cureus.83315

**Published:** 2025-05-01

**Authors:** Pochollo Miguel P Rosales, Catherine Joy Escuadra

**Affiliations:** 1 Neurosurgery, University of Santo Tomas Hospital, Manila, PHL; 2 Physical Therapy, College of Rehabilitation Sciences, University of Santo Tomas, Manila, PHL

**Keywords:** dural defect, neurosurgery, scientometric, spinal csf leak, spine surgery

## Abstract

This research aims to systematically and comprehensively evaluate the publication, citation, collaboration, and evolution of topics in dural sealants for cerebrospinal fluid (CSF) leakage in spine surgery publications. The study systematically mined publications from the Web of Science in January 2023 using the keywords (dural sealant) AND (cerebrospinal fluid) AND (spine surgery). No exclusion criteria were used in the search. The analysis was divided into publication, citation, collaboration, and text-co-occurrence network analysis. R Studio and VOSviewer were used for data management, analysis, and visualization. A total of 56 documents published between 2002 and 2022 were analyzed. The countries with the most publications were the United States of America (39.62%), Italy (9.43%), and Japan (9.43%). Total citations for all publications were 859, with an overall H-index of 15. Institutions from South Korea, India, China, and the United States of America were found to have previous collaborations on the topic. In addition, neurosurgery publications had four clusters of co-occurring keywords, which include (i) neurological deficit, polyethylene glycol, efficacy, and safety; (ii) dural defect, prevention, CSF leak; (iii) postoperative CSF leak and dural tear; and (iv) prolene, fibrin sealant, and watertight closure. In terms of evolution, publications focused initially on the types of sealants but are currently focusing more on the effects of these sealants on different outcomes. This study provided an overview of the scientific output, trends, and research topics in dural sealant for CSF leakage for spine surgery publications worldwide. More research is still needed using varied designs and more subjects on this topic. Results may serve as a valuable guide for researchers, practitioners, and educators to find the future direction of the research agenda and projects about using dural sealant to prevent CSF leak in spine surgeries.

## Introduction and background

Cerebrospinal fluid (CSF) leakage is one of the most difficult postoperative complications after spinal surgery and branches out to many further complications including epidural infections, inflammations, and an overall prolonged post-operative recovery [1]. The primary risk factors for CSF leakage include the advanced age of patients and the level of surgeon’s training, while other risk factors such as a significant history of repeated spinal surgery are also considered [2]. Since CSF leakage can result in a myriad of complications that can potentially result in a prolonged hospital stay, higher incidence of reoperation, and an overall decline in prognosis, treatment is imperative [1].

Treatment of CSF leak entails the closure of the dura to stop fluid leakage, which can be achieved by a direct suture or augmented closure, and CSF pressure adjustments [3]. While these treatment strategies have been tested for their effectiveness after incidences of CSF leakage, they also have corresponding complications. For example, direct suture repair creates pin holes upon puncture sites by the suture needles, which can alter the pressure system in the dural defect and can result in more persistent CSF leakage [3]. In addition to this, CSF pressure adjustments as a potential approach for dural repair can result in spinal fluid over drainage, entry of gas to the subarachnoid space, and entry of infectious agents [3]. With these challenges in the treatment approach for CSF leakage, dural repair calls for a more secure and airtight intervention that can also prevent the other complications of the previous approaches [3]. 

Dural sealants are biomaterials designed to provide a watertight closure of the dura mater to prevent CSF leakage after surgery [1]. These sealants typically consist of synthetic polymers such as polyethylene glycol hydrogel, fibrin-based materials, or other adhesives that rapidly polymerize to seal the defect [1]. Compared to traditional suturing techniques, dural sealants offer the advantage of faster application, reduced risk of creating additional dural defects, and better coverage of irregular tears [1]. Their mechanism involves adhering to the dural surface and forming a physical barrier, thereby restoring CSF integrity without excessive tension on the dura [4]. Advantages include ease of application, minimized manipulation of neural tissues, reduced operative time, and potential decrease in postoperative CSF leakage rates [1]. However, disadvantages include cost and the potential for inflammatory reactions in some materials [4].

At present, scientometrics has been used to understand the trend for specific neurosurgery techniques such as pallidotomy [3] and spine surgery [5], publications from certain journals [6,7], and publications of neurosurgeons from specific areas like China [8] and Africa [9]. To the authors’ knowledge at the time of writing, there is no scientometric study analyzing the use of dural sealants in the prevention of CSF leak in spine surgery. Therefore, the aims of the study are to elaborate on (i) publication and citation trends of dural sealants in spine surgery research publications, (ii) which countries and affiliations collaborate to create research publications regarding dural sealants, (iii) cluster of keywords in dural sealants in spine surgery publications using text co-occurrence analysis, and (iv) what are the areas in dural sealants and CSF leak that need further development. To expound on this matter, the objective of the study is to compare the publication trend and topic trend on dural sealants in spine surgery using scientometric techniques.

## Review

Materials and methods

Design

In this study, scientometric methods were utilized, based on the research of Gracio et al. [10] and Sangam et al. [11], to conduct a comparative analysis of the research productivity in dentistry and genetics across different countries. The use of this approach provides valuable insights into the current state and potential future directions of research, policy, education, and health related to a specific topic.

Data Source, Search Strategy, and Data Extraction

**Figure 1:**
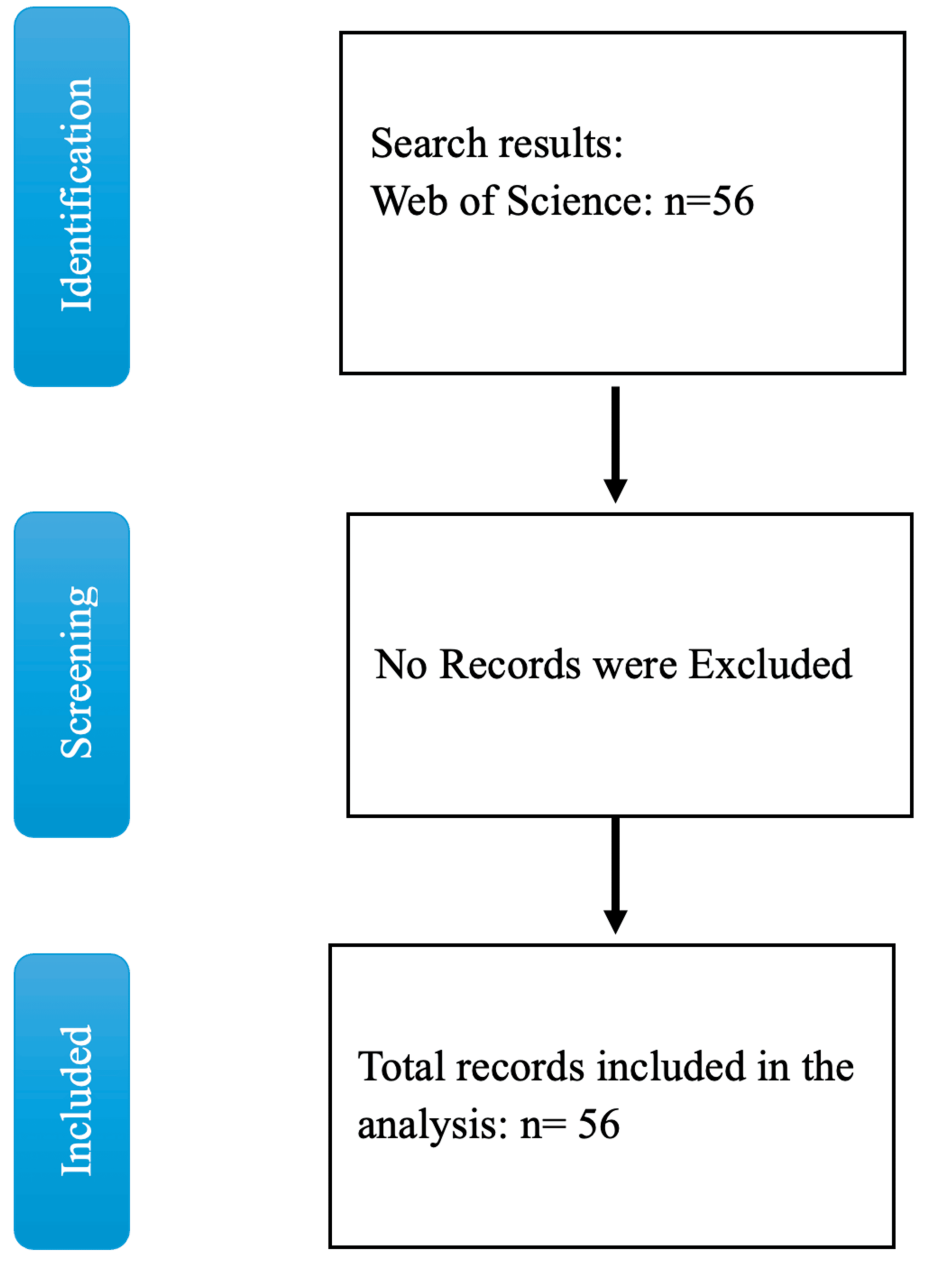
PRISMA flow diagram of the literature search PRISMA: Preferred Reporting Items for Systematic Reviews and Meta-Analyses

In January 2023, a comprehensive search was conducted of the Web of Science's core collection to retrieve pertinent articles, similar to Yao et al. [12] who utilized this database to chart the patterns and insights of global health systems research publications. The terms (“dural sealants” AND “spine surgery” AND “cerebrospinal fluid”) were used as the keywords to search the database. Complete records of each publication were extracted as plain text files from Web of Science. No exclusion criteria were used in the search as this was an exploratory study. After the initial retrieval, publications were manually categorized according to document type (original research articles, review articles, editorials) (Figure 1). More so, retrieved publications underwent a relevance screening based on title and abstract review to ensure alignment with the study objective of dural sealants in spine surgery.

Data Analysis and Visualization

In order to effectively manage and analyze the results of our research, various tools including the Web of Science result analysis feature, R Studio, and VOSviewer (version 1.6.18) were utilized. The Web of Science result analysis feature was instrumental in examining the publication and citation characteristics of the included articles, enabling us to gain a more comprehensive understanding of the data.

To further aid in the analysis, full publication and citation records were downloaded as a plain text file for text co-occurrence network analysis. This was particularly helpful in identifying connections and patterns within the data that may have otherwise gone unnoticed.

In addition to these tools, VOSviewer [13] and R Studio were employed to create various maps based on network and text data, helping us to better visualize and interpret our findings. To ensure accuracy, VOSviewer thesaurus file was utilized to merge different variants of author, journal names, institutional affiliations, and synonyms, resulting in a more cohesive and comprehensive analysis of the data.

Results

**Figure 2:**
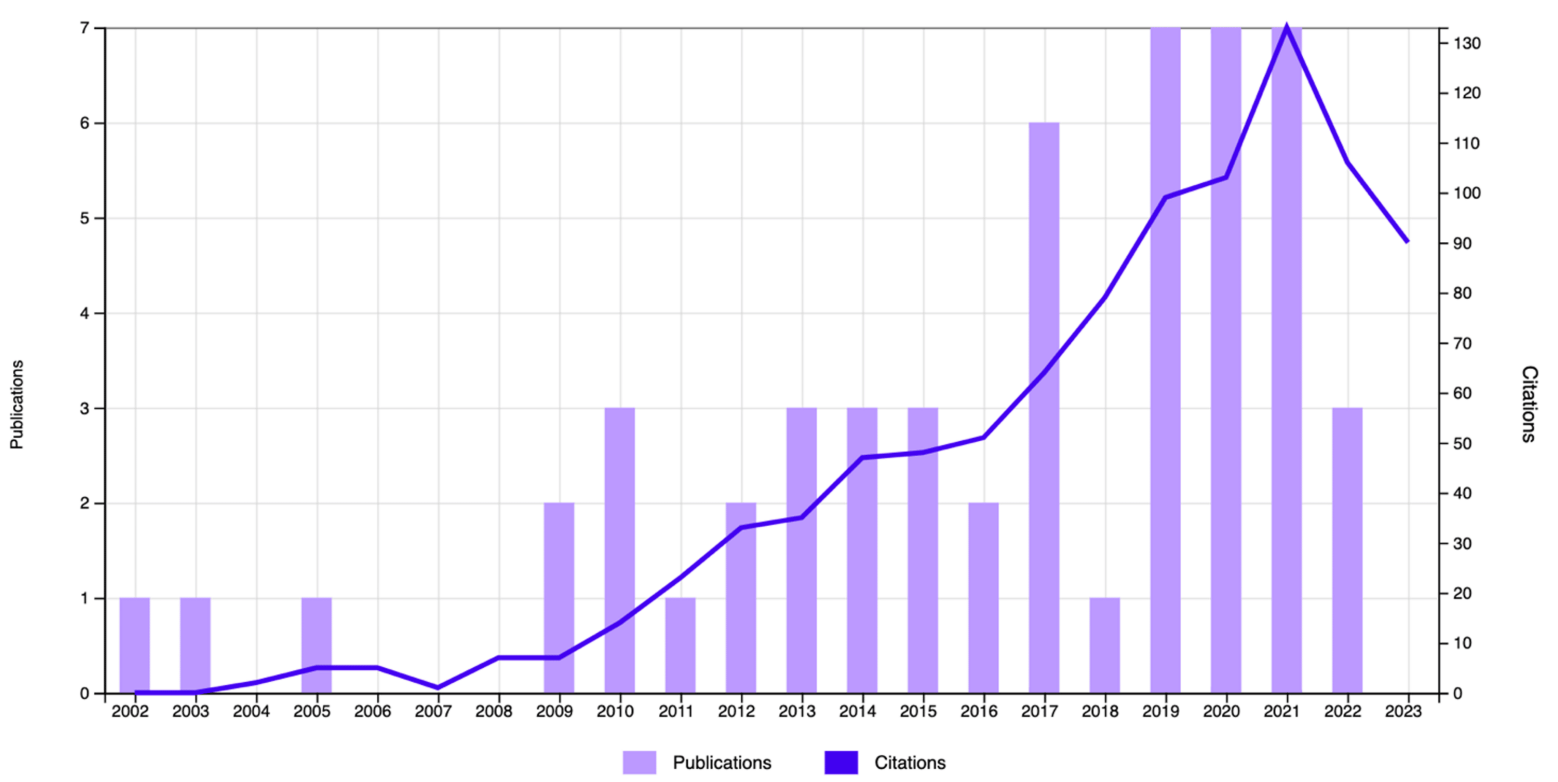
Overall publication and citation of dural sealant spine research from Web of Science

A total of 56 documents published between 2002 and 2023 were analyzed. All publications were in the English language and included the following document types: article (n=45), review (n=10), and editorial material (n=1). Total citations for all publications were 859, with an overall H-index of 15. Table 2 in Appendices lists the articles retrieved from the database. Figure 2 reports dural sealant spine research's overall publication and citation over time. 

**Figure 3:**
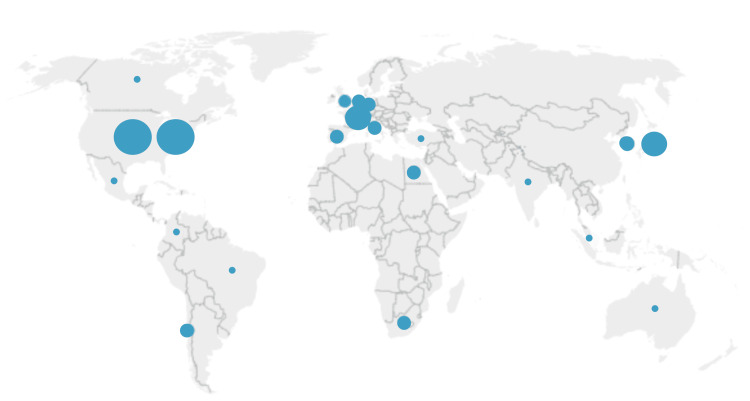
Research productivity of countries for dural sealants

Figure 3 illustrates the research productivity per country on the topic. Analysis revealed that as of 2022, 23 countries from all continents around the world have published an article about dural spine sealant. Countries with the most significant number of publications are the United States (n=22), Italy and Japan (n=5), and France, Germany, India, and South Korea (n=3). This result is further supported by the top publishing institution identified, the University of California System (n=4). In terms of evolution, the analysis revealed that the United States, India, and South Korea were the countries that initially published articles on the topic in the early 2000s, which were followed by publications from other European and South American countries. In addition, institutions from South Korea, India, China, and the United States of America were found to have previous collaborations on the topic. Also, the circle sizes in Figure 3 are proportional to the number of publications per country. The occurrence of two separate nodes for the United States was due to software limitations during thesaurus merging; this was consolidated during the final analysis to avoid duplication.

Table 1 summarizes the top three journals with the most dural sealant spine publications. Aside from these, other journals that have published articles related to this topic included journals on pediatrics (Journal of Neurosurgery Pediatrics), orthopedics (Orthopaedics Traumatology Surgery Research), and clinical neuroscience (Journal of Clinical Neuroscience). Upon further review, most of the publications have utilized the following study designs: retrospective chart review (n=15), quasi-experimental (n=13), systematic or literature review (n=10), case reports (n=10), and randomized controlled trial (n=4). All primary research used data from human subjects except for five papers that used cadavers (n=1) and animal subjects (n=4). 

**Table 1:** Top three journals with most publications on spinal dural sealant

Publication Titles	n	%	Impact Factor
World Neurosurgery	8	20.811	2.0
Spine	7	9.639	3.0
Spine Journal	5	7.01	4.16

**Figure 4:**
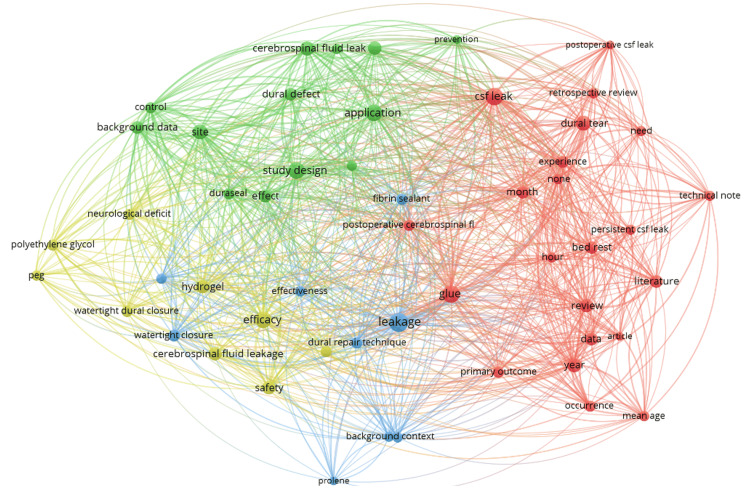
Network map of frequently occurring keywords of spinal dural sealant publications

Figure 4 presents the text map of the frequently occurring keywords in the title and abstracts of all publications included. These words were clustered together based on the frequency of their co-occurrence in each publication. Results revealed that publications in spine surgery have four clusters of co-occurring keywords which include (i) “neurological deficit,” “polyethylene glycol,” “efficacy,” “safety” (yellow); (ii) “dural defect,” “effect,” “prevention,” “cerebrospinal fluid leak” (green); (iii) “postoperative CSF leak,” “dural tear,” “review” (red); and (iv) “leakage,” “prolene,” “fibrin sealant,” “waterlight closure” (blue). These clusters of keywords were further supported by the top three most cited publications, which include “Effect of fibrin glue on the prevention of persistent cerebral spinal fluid leakage after incidental durotomy during lumbar spinal surgery” by Jankowitz et al., “Polyethylene Glycol Hydrogel Spinal Sealant (DuraSeal Spinal Sealant) as an Adjunct to Sutured Dural Repair in the Spine Results of a Prospective, Multicenter, Randomized Controlled Study” by Kim and Wright, and “Dural repair with four spinal sealants: focused review of the manufacturers' inserts and the current literature” by Epstein [4]. It is also worth noting that in terms of evolution, keywords related to primary studies like “retrospective review”, “background data,” and “effectiveness” were found in earlier publications compared with words like “literature review,” and “systematic review”. Also, keywords related to outcomes were identified like “leakage,” “primary outcomes,” and “safety.” These keyword clusters were automatically generated based on co-occurrence frequency, but occasional overlaps, such as "cerebrospinal fluid leak" and "postoperative CSF leak" in different clusters, may occur due to variations in how terms appeared in individual publications. The top three most-cited publications further validate these clusters as they primarily addressed the key concepts found across the clusters, effectiveness of dural sealants, prevention of persistent CSF leak, and safety outcomes, thus reinforcing the network analysis findings.

Discussion

This study described the publication and topic trend in dural sealant spine research in the last two decades. The movement in research publications supports this intervention's growing importance and development in spinal neurosurgery. 

Scarcity of Studies Regarding Different Strategies for Dural Repair

The study's findings indicate that the majority of current research on dural sealants in spine surgery consists of retrospective studies and case reports involving individuals who have undergone spinal surgery and there is scarcity in studies reviewing the efficacy of different strategies for dural repair. This aligns with previous reviews of spinal sealants and techniques for spinal dural tears [14]. It is worth noting, however, that recent publications on the subject include studies with higher levels of evidence, such as randomized controlled trials and systematic reviews. This development suggests that the evidence for dural sealants in spine research is growing and becoming more robust. Surgeons and researchers must prioritize the continued research of dural sealants in spine surgery to fully understand its features and effectiveness. It is crucial that we invest in this technology to improve patient outcomes and advance the field of spinal surgery.

Economic Burden of Treating CSF Leakage After Spine Surgery

Incidental durotomy and subsequent CSF leakage are established complications following spinal surgery, occurring in 2 to 20% [15]. This entails an increase in hospital expenses to as high as 50 percent. Therefore, minimizing complications during surgery with prompt repair of dural tears is important for a fast recovery and the avoidance of further complications. Hence, there is an increase in pressure on the surgeons to be careful in avoiding, as well as treating, this complication [15].

Emerging Technology and Innovations in Spine Surgery

Newer and emerging technologies, such as nanotechnology-enhanced dural sealants in the form of chitosan hydrogel, demonstrated effective sutureless dural closure, highlighting its potential in enhancing tissue integration and durability [16]. Another area of increasing interest is robot assisted applications in spinal surgery, which could enhance the precision in procedures, which could decrease incidence of complications such as CSF leak [17].

Limitation and Recommendations

As with any research endeavor, it is important to acknowledge the limitations encountered in this study. Firstly, the scope was restricted to publications indexed in the Web of Science database, potentially overlooking valuable information published in other journals. More specifically regarding the electronic search, it must be noted that the exclusive use of the keyword "dural sealant" may have limited retrieval of related studies utilizing synonymous terms like "dural patch" or "dural substitute." To gain a more comprehensive understanding of the topic, future studies may consider including a broader range of publications from diverse sources. Secondly, the text network maps in this study analyzed words occurring in the title abstract with frequency of occurrence as the primary criterion. While providing valuable insights, this approach represents a relatively fundamental analysis. Future research could benefit from using more sophisticated topic modeling techniques, like Latent Dirichlet Allocation, to enhance categorization and classification of publications. By adopting these advanced methods, researchers can achieve a more nuanced and accurate representation of the various research topics within the field of spinal surgery. Moreover, although only 56 publications met the inclusion criteria, this reflects the relatively niche focus of dural sealants in spine surgery and the strict inclusion of English language, indexed, and research-based articles.

## Conclusions

This study provided an overview of the scientific output, trends, and research topics from dural sealants for prevention of CSF leak in spine surgery. The study revealed that although the publications and citations about dural sealants have been getting considerable interest in the past few years, gaps in the publication about the topic per institution and country are still evident. Most collaboration was found between countries with high publications and advanced neurosurgery practices and training. This study provides an overview and valuable guidance for neurosurgery researchers, practitioners, educators, and policymakers to find the future direction of use of dural sealants in preventing CSF leak. The authors highly suggest spine surgeons to consider the results of the study identifying future capacity-building projects, research agendas, and policy guidelines, and possibly collaborative projects between countries, to further improve research.

## Appendices

**Table 2:** Articles retrieved from the database

Author/s Name	Article Title	Year
Taniguchi, Matsubayashi, Ikeda, et al.	Clinical Feasibility of Completely Autologous Fibrin Glue in Spine Surgery	2021
Kim, Wright	Polyethylene Glycol Hydrogel Spinal Sealant (DuraSeal Spinal Sealant) as an Adjunct to Sutured Dural Repair in the Spine Results of a Prospective, Multicenter, Randomized Controlled Study	2011
Lee, Park, Lee, Kim	Postoperative Cervical Cord Compression Induced by Hydrogel Dural Sealant (DuraSeal)	2013
Kinaci, Moayeri, van der Zwan, et al.	Effectiveness of Sealants in Prevention of Cerebrospinal Fluid Leakage after Spine Surgery: A Systematic Review	2019
Wright, Park, Tew	Spinal Sealant System Provides Better Intraoperative Watertight Closure Than Standard of Care During Spinal Surgery A Prospective, Multicenter, Randomized Controlled Study	2015
Montano, Pignotti, Auricchio, et al.	Results of TachoSil® associated with fibrin glue as dural sealant in a series of patients with spinal intradural tumors surgery. Technical note with a review of the literature	2019
Choi, Chan, Brown, et al.	Effectiveness of Repair Techniques for Spinal Dural Tears: A Systematic Review	2021
Goodwin, Recinos, Zhou, et al.	Evaluation of complication rates of pediatric spinal procedures in which a polyethylene glycol sealant was used	2014
Montano, Giordano, Caccavella, et al.	Hemopatch® with fibrin glue as a dural sealant in cranial and spinal surgery. A technical note with a review of the literature	2020
Dafford, Anderson	Comparison of dural repair techniques	2015
Gazzeri, Galarza, Callovini	Use of tissue sealant patch (TachoSil) in the management of cerebrospinal fluid leaks after anterior cervical spine discectomy and fusion	2023
Al Jammal, Wali, Lewis, Zaldana, et al.	Management of Giant Sacral Pseudomeningocele in Revision Spine Surgery	2020
d'Avella, Fazzolari, Schiariti, et al.	Common Practice in the Management of Dural Closure: An Italian Questionnaire	2019
Thomas, Shetty, Rajasekaran	Abducens Nerve Palsy Associated With Pseudomeningocele After Lumbar Disc Surgery A Case Report	2012
Black	Cerebrospinal fluid leaks following spinal surgery: use of fat grafts for prevention and repair - Technical note	2002
Bakhsheshian, Strickland, Patel, et al.	The use of a novel perfusion-based cadaveric simulation model with cerebrospinal fluid reconstitution comparing dural repair techniques: a pilot study	2017
Mitchell, Verla, Reddy, et al.	Reliable Intraoperative Repair Nuances of Cerebrospinal Fluid Leak in Anterior Cervical Spine Surgery and Review of the Literature	2016
van Doormaal, Germans, Sie, et al.	Single-Arm, Open-Label, Multicenter Study to Evaluate the Safety and Performance of Dura Sealant Patch in Reducing Cerebrospinal Fluid Leakage Following Elective Cranial Surgery: The ENCASE Trial Study Protocol	2020
Agulnick, Cohen, Epstein	Unique Bone Suture Anchor Repair of Complex Lumbar Cerebrospinal Fluid Fistulas	2020
Barber, Sofoluke, Reardon, et al.	Full Endoscopic Repair of Spontaneous Ventral Cerebrospinal Fluid Leaks in the Spine: Systematic Review of Surgical Treatment Options and Illustrative Case	2022
Oitment, Aref, Almenawar, Reddy	Spinal Dural Repair: A Canadian Questionnaire	2018
Kaliya-Perumal, Lu, Kao, et al.	Delayed extensive lumbar sub-dural effusion following discectomy - Clinical imaging and case report	2017
Makia, Alawamry, Elsharkawy	Posterior and postero-lateral incidental durotomy during lumbar spine surgery: primary repair versus augmented primary repair	2021
Hughes, Ozgur, German, Taylor	Managing The Cerebrospinal Fluid Leaks After Spinal Surgery By Prolonged Subfascial Drainage	2013
Andrew, Sidhu	Cervical-peritoneal shunt placement for postoperative cervical pseudomeningocele	2005
Eser, Hanalioglu, Cetiner, et al.	Identification of Risk Factors for Postoperative Cerebrospinal Fluid Leakage and Comparison of Two Alternative Dural Augmentation Techniques in Posterior Fossa and Spinal Surgeries	2019
Epstein	Hemostasis and other benefits of fibrin sealants/glues in spine surgery beyond cerebrospinal fluid leak repairs	2014
Epstein	Medicolegal corner (spine): Contraindicated use of DuraSeal in anterior cervical spine led to quadriplegia	2021
Oertel, Burkhardt	Full endoscopic treatment of dural tears in lumbar spine surgery	2017
Kinaci, van Thoor, Redegeld, et al.	Ex vivo evaluation of a multilayered sealant patch for watertight dural closure: cranial and spinal models	2021
Ohtonari, Noguchi, Nishihara	Acute-Phase Intraoperative Findings of Traumatic Spinal Cerebrospinal Fluid Leakage	2021
Kogias, Klingler, Franco Jimenez, et al.	Incidental Durotomy in Open Versus Tubular Revision Microdiscectomy: A Retrospective Controlled Study on Incidence, Management, and Outcome	2017
Nowak, Schroeder, Fleck	Hemopatch® as a new dural sealant: A clinical observation	2019
Takenaka, Makino, Sakai, et al.	Prognostic impact of intra- and postoperative management of dural tear on postoperative complications in primary degenerative lumbar diseases	2019
Kulkarni, Tapashetti, Patel	Use of Zipper Ratcheting Straps as a Bailout for Postoperative Cerebrospinal Fluid Leaks: Our Experience	2020
Fountas, Kapsalaki, Johnston	Cerebrospinal fluid fistula secondary to dural tear in anterior cervical discectomy and fusion - Case report	2005
Slot, Bergmann, Kinaci, et al.	Histological and magnetic resonance imaging assessment of Liqoseal in a spinal in vivo pig model	2023
Moussa, Aboul-Enein	Combined thrombin and autologous blood for repair of lumbar durotomy	2016
Koechlin, Burkhardt, Scherer, et al.	Cerebrospinal Fluid Leaks after Planned Intradural Spine Surgery: a Single-Center Analysis of 91 Cases	2013
Serikyaku, Higa, Yara	Intradural disc herniation at the L1-2 level	2021
Jankowitz, Atteberry, Gerszten, et al.	Effect of fibrin glue on the prevention of persistent cerebral spinal fluid leakage after incidental durotomy during lumbar spinal surgery	2009
Jeon, Lee, Tsang, et al.	Watertight Sealing Without Lumbar Drainage for Incidental Ventral Dural Defect in Transthoracic Spine Surgery A Retrospective Review of 53 Cases	2017
Alshameeri, El-Mubarak, Kim, Jasani	A systematic review and meta-analysis on the management of accidental dural tears in spinal surgery: drowning in information but thirsty for a clear message	2020
Han, Jeong, Kim, Seung	Postoperative Thecal Sac Compression Induced by Hydrogel Dural Sealant after Spinal Schwannoma Removal	2020
Khatri, D'Amico, Tucker, et al.	Surgical Management of Symptomatic Boxing-Induced Spinal Cerebrospinal Fluid Leak After Failed Epidural Blood Patch	2020
Mulder M, Crosier J, Dunn R	Cauda Equina Compression by Hydrogel Dural Sealant After a Laminotomy and Discectomy Case Report [46]	2009
Hida, Yamaguchi, Seki, et al.	Nonsuture dural repair using polyglycolic acid mesh and fibrin glue: Clinical application to spinal surgery	2006
Preul, Campbell, Garlick, Spetzler	Application of a new hydrogel dural sealant that reduces epidural adhesion formation: evaluation in a large animal laminectomy model Laboratory investigation	2010
Miscusi, Polli, Forcato, et al.	The use of surgical sealants in the repair of dural tears during non-instrumented spinal surgery	2014
Theys, Van Hoylandt, Broeckx, et al.	Plasma-rich fibrin in neurosurgery: a feasibility study	2018
Xiong, Tobert, Fogel, et al.	Open epidural blood patch to augment durotomy repair in lumbar spine surgery: surgical technique and cohort study	2021
Wolff, Kheirredine, Riouallon	Surgical dural tears: Prevalence and updated management protocol based on 1359 lumbar vertebra interventions	2012
Epstein	Wound-peritoneal shunts: part of the complex management of anterior dural lacerations in patients with ossification of the posterior longitudinal ligament	2009
d'Astorg, Szadkowsk, Vieira, et al.	Management of Incidental Durotomy: Results from a Nationwide Survey Conducted by the French Society of Spine Surgery	2020
Das, Goyal, Hunt	Intrathoracic meningocele associated with neurofibromatosis Type 1 and a novel technique for surgical repair: case report	2017
Thavara, Kidangan, Rajagopalawarrier	Analysis of the Surgical Technique and Outcome of the Thoracic and Lumbar Intradural Spinal Tumor Excision Using Minimally Invasive Tubular Retractor System	2019
